# Developing a hybrid dictionary-based bio-entity recognition technique

**DOI:** 10.1186/1472-6947-15-S1-S9

**Published:** 2015-05-20

**Authors:** Min Song, Hwanjo Yu, Wook-Shin Han

**Affiliations:** 1 Department of Library and Information Science, Yonsei University, South Korea; 2 Department of Computer Science and Engineering, POSTECH, Pohang, South Korea; 3 Department of Creative IT Engineering, POSTECH, Pohang, South Korea

## Abstract

**Background:**

Bio-entity extraction is a pivotal component for information extraction from biomedical literature. The dictionary-based bio-entity extraction is the first generation of Named Entity Recognition (NER) techniques.

**Methods:**

This paper presents a hybrid dictionary-based bio-entity extraction technique. The approach expands the bio-entity dictionary by combining different data sources and improves the recall rate through the shortest path edit distance algorithm. In addition, the proposed technique adopts text mining techniques in the merging stage of similar entities such as Part of Speech (POS) expansion, stemming, and the exploitation of the contextual cues to further improve the performance.

**Results:**

The experimental results show that the proposed technique achieves the best or at least equivalent performance among compared techniques, GENIA, MESH, UMLS, and combinations of these three resources in F-measure.

**Conclusions:**

The results imply that the performance of dictionary-based extraction techniques is largely influenced by information resources used to build the dictionary. In addition, the edit distance algorithm shows steady performance with three different dictionaries in precision whereas the context-only technique achieves a high-end performance with three difference dictionaries in recall.

## Background

### Introduction

The extraction of biomedical entities from scientific literature is a challenging task encountered in many applications such as system biology, molecular biology, and bioinformatics. One of the early, continuously adopted approaches is the dictionary-based entity extraction. Dictionary-based entity extraction extracts all the matched strings from a given text by entities defined in a dictionary. Based on the lemma for a given term, it recognizes a term by searching the most similar (or identical) one in the dictionary. This makes dictionary-based approaches particularly useful for practical information extraction from biomedical documents as the first step for extraction [6]. In addition, dictionary-based approaches are very useful when there are no or minimal contexts available to detect named entities such as a query.

However, dictionary-based approaches have two major performance bottlenecks. First, the false positives, inherent with using short names, significantly degrade the overall accuracy. Exclusion of short names from the dictionary may resolve this issue, but it is not the ultimate solution in that such a solution disallows for recognizing short protein or gene names. Second, spelling variation makes dictionary-based approaches less usable. For example, the gene name "DC2-dopamine receptor" has many spelling variants such as "dopamine DC2 receptor," and "dopamine DC2 receptor." Exact matching techniques mainly employed by dictionary-based approaches treat these terms as distinct ones.

We alleviate this problem by using an approximate string matching method in which surface-level similarities between terms are considered. In order to mitigate the low recall problem associated with dictionary-based approaches, we combine entity extraction with soft-matching scheme that is capable of handling variant entity names. To this end, we propose a new entity extraction technique comprised of several different techniques. The proposed technique consists of 1) the approximate string distance algorithm to retrieve candidate entries, 2) shortest-path edit distance algorithm (SPED), and 3) text mining techniques such as Part-Of-Speech (POS) tagging and utilization of syntactical properties of terms. The experimental results show that in most cases, the performance of the proposed technique is superior to other approaches.

The rest of the paper is organized as follows: Section 2 describes the studies related to the present paper. Section 3 explains the proposed technique in depth. Section 4 reports on the data collection and the experimental results. Section 5 concludes the paper with a discussion of future research.

### Related works

The dictionary-based entity extraction is still widely used method for biomedical literature annotation and indexing [13]. The major advantages of dictionary-based technique over the pattern-based approach are twofold: it allows for recognizing names and identifying unique concept identities. The exact match approach is the simplest one; however, it suffers from low recall due to the ingrained variants (morphological, syntactic, and semantic) characteristic of a biological term (Chiang and Yu, 2005). In addition, it is nearly impossible for a dictionary to collect them all. One entity type extraction, combining dictionary-based with supervised learning techniques, dictionary Hidden Markov Models (HMMs) represent a technique in which a dictionary is converted to a large HMM that recognizes phrases from the dictionary, as well as variations of these phrases [1].

Stemming from the development of the GENIA corpus [9], many studies have explored extraction tasks including "protein," "DNA," "RNA," "cell line," and "cell type" (e.g., [11,10]). In addition, some studies have targeted "protein" recognition only [12]. Other tasks include "drug" [13] and "chemical" (Narayanaswamy et al. [3]) names. Another related research area related to entity mapping is semantic category assignment. Most of the work about semantic category assignment is done in the context of named entity tagging where terms in the text will be assigned categories from a list of predefined categories. Features for semantic category assignment include both words within a phrase and contextual features derived from neighbouring words. In the general English domain, Frantzi et al. [14] used term similarity measures based on phrase-inner and contextual information (C/NC-values), where the similarity measure for phrase-inner clues was used to distinguish headwords from modifiers. For language independent named entity recognition(first name, last name, and location), Cucerzan and Yarowsky [15] proposed a trie-based approach to combine name affix information and contextual information, where affix information is informative in detecting names, while names can be ambiguous among the name classes. In the biomedical domain, words within a phrase tend to be more effective since most biomedical terms are descriptive noun phrases. Many systems depend on a set of manually collected headwords or suffixes for semantic category assignment [16-18].

Besides hand-crafted methods, machine learning methods have also been explored in the domain of extract bio-entities. Nenadic et al. [4] used a method similar to the C/NC-values [14] to identify similar terms. Hatzivassiloglou and colleagues [19] used a method similar to our two-step corpus-based learning for the disambiguation of protein, gene, or RNA class labels for biological terms in free text. Nobata et al. [20] created a system that assigns four semantic categories (i.e., protein, DNA, RNA, and source) based on supervised machine learning systems trained on 100 MEDLINE abstracts. Similarly, Lee et al. [21] developed a two-phase name recognition system, where separate detection and classification modules were developed using support vector machine. They used different and specialized sets of features for each task, and obtained better results than those of the one-phase model over the GENIA corpus annotated with 23 categories. The NER technique has also been applied to extract chemical components from text. ChemSpot is the NER algorithm that extracts mentions of chemicals such as trivial names, drugs, abbreviations, and molecular formulas [23] whereas [24] focuses on the identification of International Union of Pure and Applied Chemistry (IUPAC) chemical compounds. Drug-drug interaction is another application area of NER. DDIExtraction 2013 is the extraction task that concerns the recognition of drugs and extraction of drug-drug interactions that appear in biomedical literature [24].

## Methods

In this section, we provide in-depth details of the proposed technique. The algorithm is described in Table 1.

**Table 1:** Algorithm of The proposed technique.

Given a dictionary Input: short passage
1: Apply the approximate string matching technique 2: Generate candidate matched entries **For each **generated entry list 3: Apply the shortest path edit distance (SPED) technique 4: **If **there is a perfect match between the input and the matched entry, exit the loop 5: **Else** 6: Merge the candidate list by the context-enabled text mining techniques 7: Select the best merged entry 8: Return the matched entity type

### Dictionary construction

**Figure 1:**
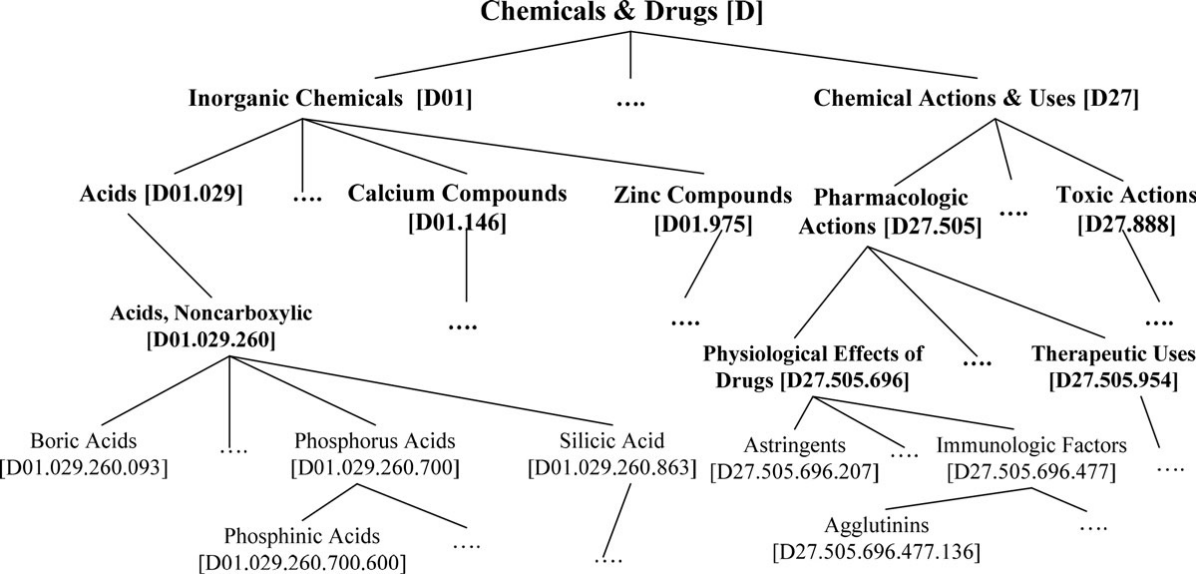
**Portion of MeSH tree hierarchy**.

We utilized the Medical Subject Headings (MeSH) tree as one of the sources to construct an entity mapping dictionary. MeSH contains 25,588 term descriptors (2010 version) that denote various general medical terms such as Anatomy Disorders and Physiologic Function, as well as specific terms such as B-Lymphocytes, Regulatory and Langerhans Cells. MeSH is managed by domain experts and indexers at the National Library of Medicine (NLM). Their primary task is to assign the most specific terms in the MeSH tree to a PubMed record. MeSH terms are structured in a tree hierarchy that defines the hierarchical relationship among terms. Figure 1 presents a portion of MeSH that describes Phosphinic Acids and related acids. The hierarchy is represented by a tree of nodes, with one or several nodes mapping to a single term label. For example, the term Rheumatic Diseases is represented by the top category which is Diseases.

Descriptors provided in MeSH are grouped into 16 categories. For example, category A, B, C, and C are related to anatomic terms, organisms, diseases, drugs and chemicals, respectively. Each category consists of an array of subcategories, and in each subcategory, descriptors are structured in the form of an upside down tree. Although MeSH categories do not officially represent an authoritative subject classification scheme, they help guide indexers to assign subject headings to documents or researchers to search for literature. In this paper, MeSH tree enables us to build a dictionary where each MeSH term in the sub-trees is mapped to the top label term. To expand the idea of the dictionary construction, we make use of GENIA as well as UMLS to examine the influence of the source on mapping accuracy.

GENIA is a gene corpus consisting of 1,999 MEDLINE records to help develop and evaluate information extraction and text mining systems for the medical domain. The corpus is annotated with various levels of linguistic and semantic information related to genes. UMLS stands for Unified Medical Language System developed by the National Library of Medicine. UMLS consists of three knowledge sources: the Metathesaurus, the Semantic Network, and the SPECIALIST Lexicon. The Metathesaurus is a vocabulary database that contains information about biomedical related concepts and the relationships among them. The semantic network categorizes all concepts represented in the UMLS Metathesaurus and provides a set of useful relationships between these concepts. The SPECIALIST Lexicon provides the lexical information needed for the SPECIALIST NLP tool.

### Approximate string matching technique

The exact match technique is the simplest one to utilize a dictionary to spot candidate terms. Several fast exact match algorithms such as Boyer-Moore algorithm [5] have been proposed. However, spelling variations make the exact match impractical less attractive. For example, a protein name "EGR-1" has the following variations: Egr-1, Egr 1, egr-1, egr 1, EGR-1, and EGR 1.

Unlike this exact matching algorithm, the approximated matching technique is based on weighted edit distance of strings from dictionary entries. In other words, it is a fuzzy dictionary matching strategy. The data structure for the underlying dictionary is a trie in order to support efficient search for matches. The approximate string matching technique is implemented based on the algorithm proposed by [12]. The algorithm consists of two phases: 1) finding approximate substring matches inside a given string and 2) finding dictionary strings that match the pattern approximately. The closeness of a match is measured by a set of operations of edit distance to convert the string into an exact match. For example, in the case of the term "E coli," this term is compared against the dictionary constructed by an approximate string match technique, and a matched entry for "E coli," "Escherichia coli Proteins," is found in the dictionary once the threshold is set.

### Shortest Path Edit Distance (SPED)

SPED, an extension of algorithm from Rudniy et al. [7], calculates edit distance between two strings, at character level.

**Figure 2:**
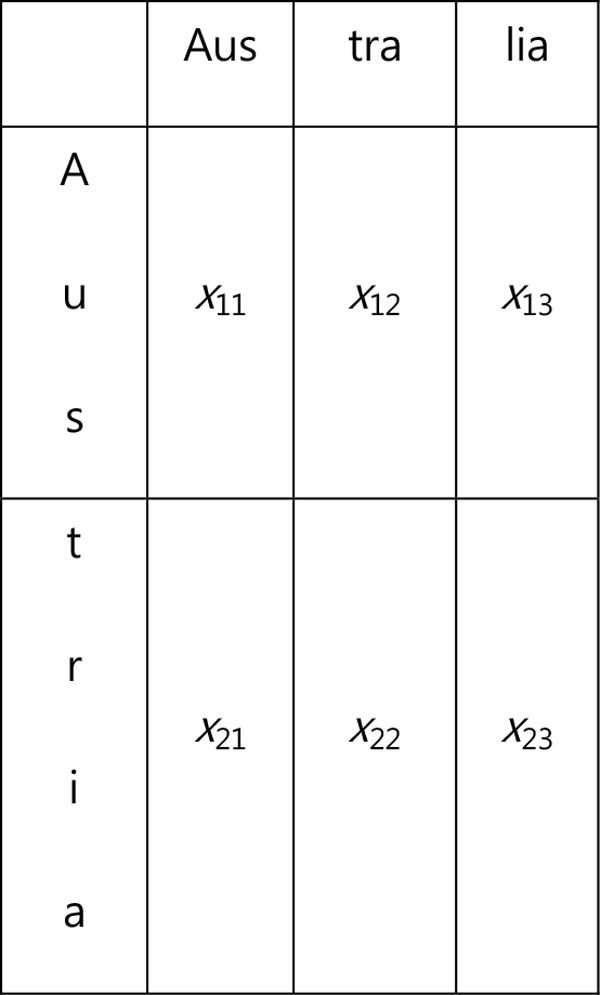
**Sample string alignment**.

The goal of the SPED algorithm is to calculate a string distance score for a pair of strings. The score shows how similar or dissimilar these strings are. Two strings *S *and *T *are aligned on the sides of a rectangular. String S is placed along the top horizontal side while string T is aligned along the left vertical side. The two strings are divided into the substrings of the same length *L*, which is an adjustable parameter. The last substring of *S *or *T *may be longer when their lengths are not a multiple of *L*. The rectangle is divided into boxes. Each box is assigned to the pair of corresponding substrings of S and T as shown on Figure 2.

For each box, a score *x_ij _*is computed by formula (1).

(1) $$\begin{array}{c} x_{ij}=\frac{\overset{l}{\underset{a=1}{\sum }}\overset{l}{\underset{b=1}{\sum }}MatchScore\left(S\left[a\right],T\left[b\right]\right)}{l^{2}}\\ MatchScore\left(S\left[a\right],T\left[b\right]\right)=\left\{\begin{array}{cc} 0, & S\left[a\right]=T\left[b\right]\\ 1, & S\left[a\right]\neq T\left[b\right] \end{array}\right) \end{array}$$

**Figure 3:**
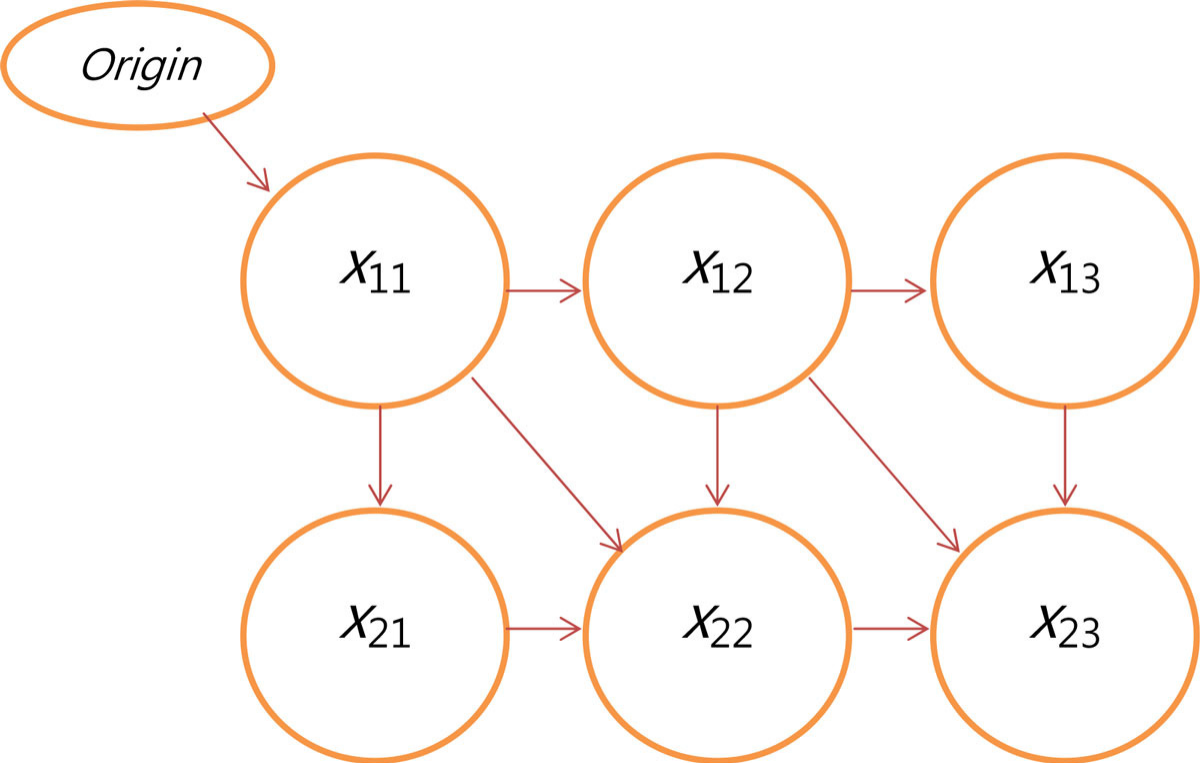
**Weighted directed acyclic graph**.

Then, the grid is transformed into a weighted directed acyclic graph, where each box becomes a graph node. The graph is shown in Figure 3.

The incidents edges on each node are assigned the weight of the corresponding node. Then, by applying the Pulling algorithm, the shortest path between the origin and the bottom right node is computed, accordingly. To normalize the distance, the weight of the path is divided by the number of edges traversed. Now, two strings are checked for the presence of a common prefix. If such a prefix is found, the final score is adjusted by a re-scorer:

(2) $$SPED=SPED^{\prime }-\left|Prefix\right|*0.1*\left(1-SPED^{\prime }\right)$$

Where SPED is the final value of the string distance, |*Prefix*| is the length of the common prefix, *SPED*' is the normalized weight of the shortest path.

### Lattice of neighbourhood of string

The SPED algorithm's tasks are twofold; firstly, identification of the shortest path from a directed weighted acyclic graph, secondly, computation of the edit distance among strings. We construct nodes lattice, which represents substring interactions s[(i-k)...i]s[(i-k)...i]and t[(j-k)...j] where k=0...min(n,m); the strings lengths, s and t, are represented by n and m, respectively. Let Neighborhood of String (NS) be a set, C, of consequent substrings each having length k, where k = 1,..., n, for $\left\lfloor n/k\right\rfloor$ elements, and the length of $\left\lceil n/k\right\rceil$-th element is $n-\left\lfloor n/k\right\rfloor \cdot k$. Furthermore, a numeric value in the range, 1,...,$\left\lceil n/k\right\rceil$, is assigned to each neighborhood.

The NS interaction among two strings defines a lattice element. The NS edit operation result into value, which is assigned to a lattice node. The edit operation results into a weight value, corresponding to a pair of NSs; which is the transformation cost of converting a substring from the first string into a substring from the second string. Five different methods are taken into consideration for weight assignment, which are used and tested in the experiments.

### Lattice-based graph composition

**Figure 4:**
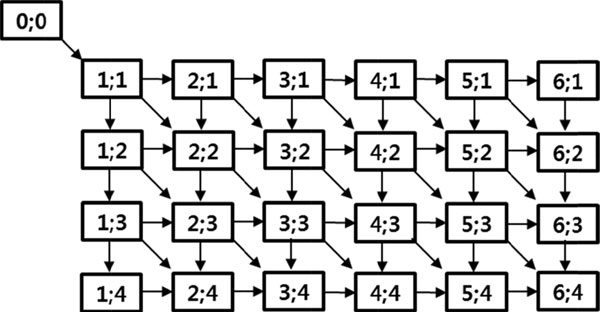
**Graph constructed from the 6x4 lattice**.

After the lattice is constructed, it is transformed into a graph, which is weighted, directed, and acyclic, see Figure 4. In edit distance operation, if two strings match, they are placed into the vertical and horizontal sides of the matrix; consequently, the matrix is populated as a result of each edit-distance operation. The edit path constitute a cells sequence, from the cell that denote the first character of each string to the cell that is located at the intersection of the last characters, where the last cell contains result of edit distance operation. The first cell can be moved to the last cell, horizontally (for deletion), vertically (for insertion), or diagonally (for substitution or two characters match).

In this paper, the aforementioned idea is applied by converting each lattice cell to a graph vertex. A source vertex is added at the left top side and connected to the vertex (1; 1) of the graph by a diagonal edge. A gap cost is assigned to edges, which is selected during a learning phase. As discussed earlier, a weight value is assigned to each diagonal edge; this weight value is a result of an NS edit operation, which is stored in the lattice cell. Since the source vertex is a placeholder, it is used as a starting point by the algorithm. In the SPED algorithm, the calculation of a string distance value between two strings becomes the shortest path calculation task from the source to the destination vertices. The destination vertex corresponds to the pair of last NSs of strings S and T. The graph is a directed, weighted, and acyclic.

### Merging strategy

To handle the issue of similar concepts, we combine them into a representative one by the following rule: The shorter term is merged into the longer term when 1) the starting position of both terms is identical, 2) they have the same top category - contextual cues, 3) they are either noun term or phrases - POS tagging, and 4) they share similar lexical properties.

## Results

### Data collection

To measure the performance of the proposed technique in a comprehensive manner, we used three different data sources: 1) GENIA, 2) Mesh Tree, and 3) UMLS. GENIA ontology is a taxonomy of 47 biologically relevant nominal categories [9]. GENIA corpus consists of 96 582 annotations. Among them, 89 682 are for surface level terms, 1583 are for higher level terms. As described earlier, we used the MeSH Tree Structure that organizes 25,588 MeSH concepts under the 16 top categories. UMLS is the most well-received ontology in the biomedical domain. It consists of 2,918,970 concepts.

For evaluation of the proposed techniques, we used JNLPBA 2004 dataset. JNLPBA 2004 data consists of 33,306 biomedical entities [8]. In the same partitioned sets, we used three different test sets for evaluation (Table 2). In JNPBA 2004 datasets, we used three subsets: 1978-1989 set consisting of 104 MEDLINE abstracts called "A" set in Table 2, 1990-1999 set consisting of 106 MEDLINE abstracts called "B" set in Table 2, 2000-2001 set consisting of 130 MEDLINE abstracts called "C" set in Table 2.

**Table 2:** Basic Statistics of the Test Data.

NER		GENIA		GENIA+MeSH		GENIA+MeSH+UMLS	
		
		Precision	Recall	Precision	Recall	Precision	Recall
The proposed technique	A	98.7%	71.4%	83.3%	71.4%	70.7%	68.9%
	
	B	94.8%	62.4%	90.3%	76.9%	90.1%	74.1%
	
	C	93.0%	57.3%	87.8%	72.0%	88.3%	68.4%

Context Only	A	69.4%	68.5%	39.6%	80.7%	33.8%	74.9%
	
	B	75.9%	66.7%	55.0%	84.8%	50.5%	82.6%
	
	C	76.4%	62.2%	56.8%	81.0%	51.7%	78.2%

SPED Only	A	98.7%	44.1%	94.4%	60.6%	94.8%	63.4%
	
	B	99.3%	41.8%	94.3%	71.7%	94.5%	72.9%
	
	C	97.6%	37.5%	93.2%	64.1%	93.6%	66.7%

As the evaluation measure, we used precision, recall and F1. We also adopted 10-fold cross validation and reported the average value of 10 trials. Precision is defined as the percentage of true positives over the total number of positives predicted by the system (Precision=TP/(TP+FP) where TP denotes the number of true positives and FP denotes the number of false positives). Recall is defined as the percentage of the number of true positives over the total number of positives in evaluation entries (Recall=TP/(TP+FN) where FN is the number of false negatives). The F1 score is the ensemble of precision and recall and defined as the inverse of the arithmetic mean of the reciprocal values of precision and recall.

### Experimental results

Table 3 shows the experimental results of the performance of the proposed technique. The evaluation is conducted in two major parts. The first evaluation focuses on the impact of data sources for dictionary construction on the performance of entity mapping. The results show that as more data sources are combined, precision drops and recall increases in most cases.

**Table 3:** Experimental results of three different combinations of the proposed technique.

Test Set	# of abstracts	# of tokens
A (1978-1989)	104	22,320

B (1990-1999)	106	25,080

C (2000-2001)	130	33,380

The second evaluation is whether and how sub components of the proposed technique (context only, SPED only, and combination of these two) have an impact on the performance. In particular, in terms of precision, the performance of entity extraction based on context-enabled text mining (described in the Merging Strategy section) gets significantly worse whereas the performance of entity extraction based on SPED does not change much. In the case of the proposed technique (which combines context and SPED), the performance drop is moderate. In terms of recall, the more data sources are combined, the better performance is observed. This is an expected outcome in that the dictionary size correlates with the performance of dictionary-based entity extraction. One interesting observation is that the performance of the proposed technique on the test set A does not increase when the dictionary is based on the combination of three sources compared to one source (GENIA). We are currently undertaking a close investigation of possible causes for this outcome.

**Figure 5:**
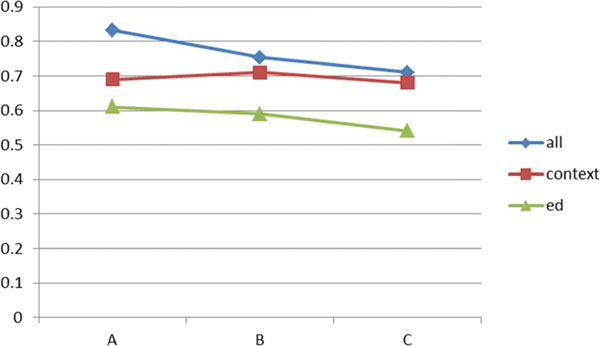
**Performance comparison on GENIA data (F-measure)**.

The performance is also measured by F1. As shown in Figure 5, the proposed technique outperforms the other two: context only and SPED. In Figure 5, "all" denotes integration of context and edit distance methods. "Context" denotes the context only method. "Ed" denotes the edit distance only method. In particular, the test dataset A shows a significant difference between the proposed technique and the other two.

**Figure 6:**
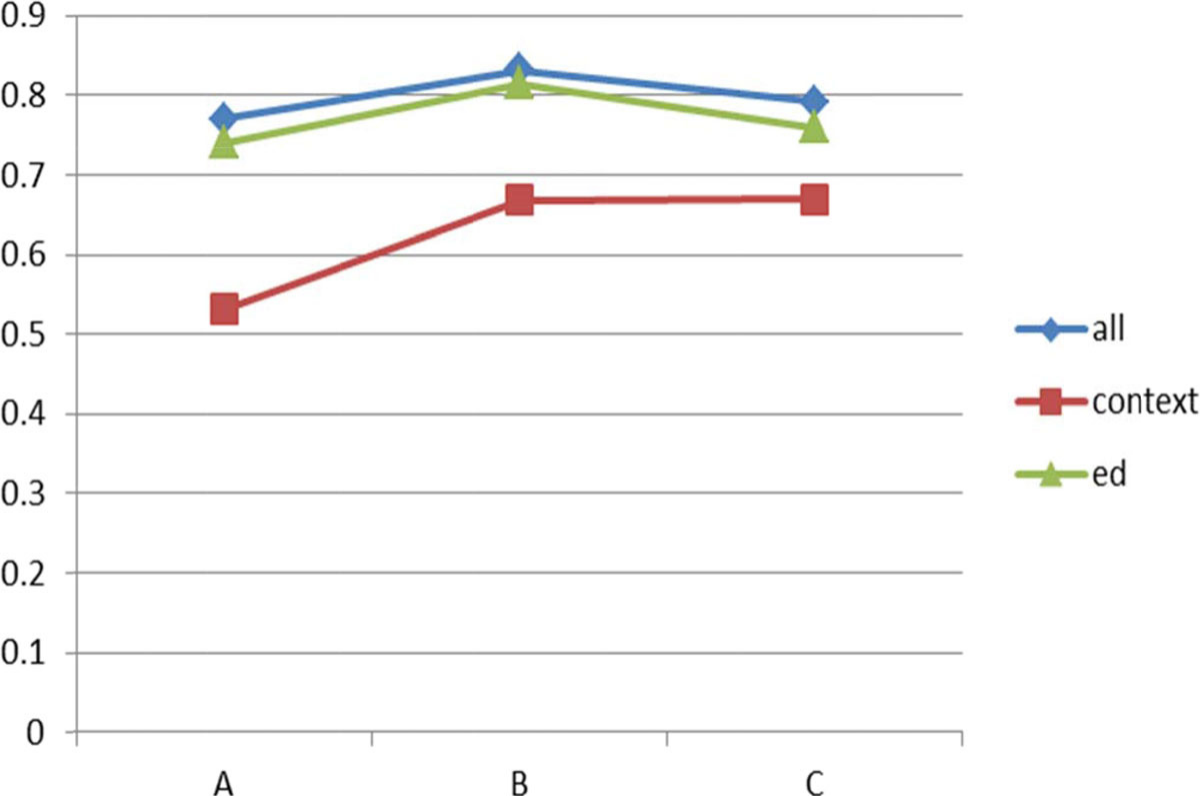
**Performance comparison on GENIA+MeSH data (F-measure)**.

Figure 6 shows the performance comparison among the three approaches on the dictionary with GENIA+MeSH. The proposed technique is slightly better than SPED in all three datasets whereas the context only option shows the worst performance overall.

**Figure 7:**
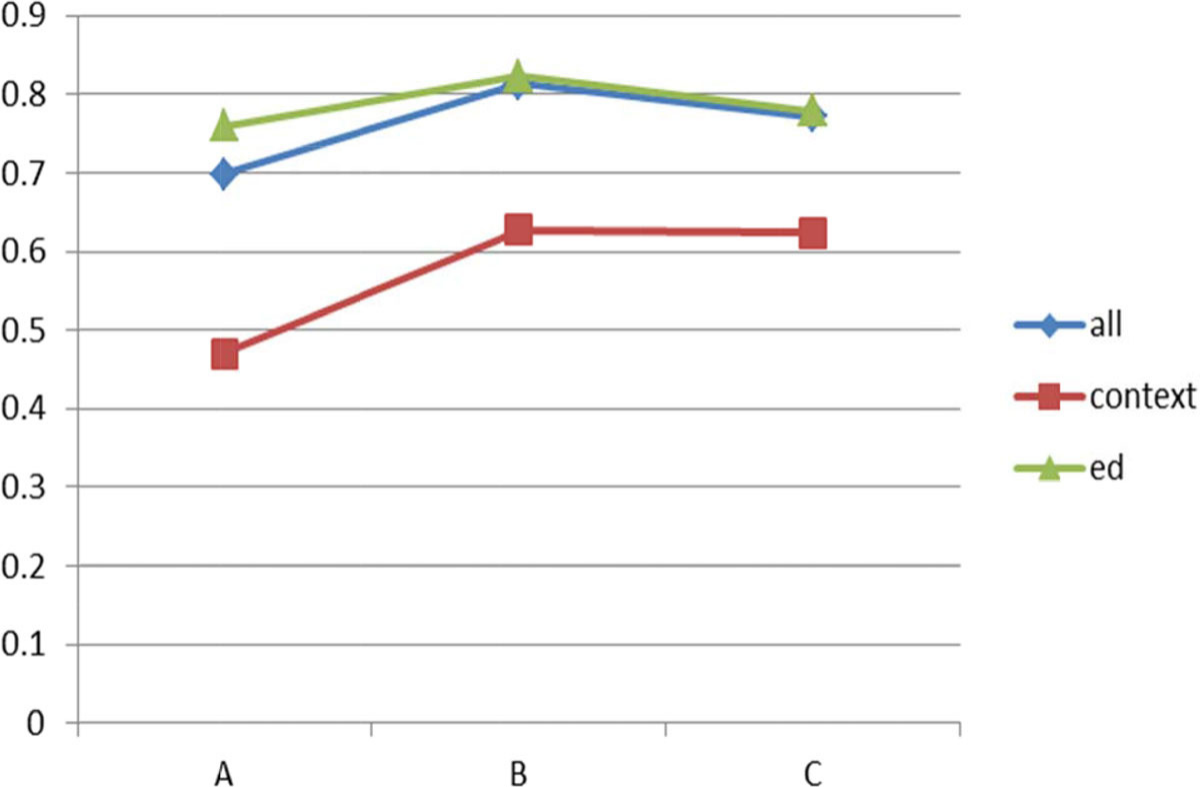
**Performance comparison on GENIA+MeSH+UMLS data (F-measure)**.

Figure 7 shows the performance comparison among the three approaches on the dictionary constructed by the combination of GENIA+MeSH+UMLS sources.

As shown in Figure 6, SPED outperforms the proposed technique on the test dataset A and has equivalent performance on the test datasets B and C. Even if the proposed technique makes a weak performance in the combination of the three sources, the overall experimental results show that the proposed technique is superior to the other two approaches in almost all cases.

In addition, we compare the proposed technique with other techniques reported in the literature. We choose the proposed technique with the dictionary constructed by the combination of the three data sources. In the conference of JNLPBA, the best performance is achieved by Zhou and Su's technique [22].

As shown in Table 4, the proposed technique outperforms Zhou and Su's technique on the test datasets B and C in the F measure whereas it is slightly inferior to [22] on the test dataset A.

**Table 4:** Performance comparison between the proposed technique and Zhou and Su's Technique (P, R, and F denote precision, recall, and F-measure respectively).

Techniques		A (1978-1989)	B (1990-1999)	C (2000-2001)
The proposed technique	P	70.7	90.1	88.3
	
	R	68.9	74.1	68.4
	
	F	69.8	81.3	77.1
	

Zho04	P	75.3	77.1	75.6
	
	R	69.5	69.2	71.3
	
	F	72.3	72.9	73.8

A series of experiments show that the performance of dictionary-based extraction techniques is largely influenced by the information resources used to build the dictionary. In addition, the edit distance algorithm shows a steady performance with the three different dictionaries in precision whereas the context only technique achieves high-end performance with those dictionaries in recall.

## Conclusions

This paper proposed a hybrid dictionary-based entity extraction technique. The proposed technique consists of 1) an approximate string matching technique, 2) a shortest path edit distance technique, and 3) context-enabled text mining techniques.

The novel feature of our method lies in the two-level string matching technique where SPED is applied to candidate sets of matched entries from a dictionary. We conducted comprehensive evaluation of the proposed technique on the JNLPBA 2004 test data. We examined the impact of the dictionary on the performance by combining three different data sources: GENIA, MeSH, and UMLS. The experimental results show that the proposed technique outperforms the approaches with text mining techniques only as well as with SPED only by F measure in most cases. In addition, the experimental results show that the proposed technique performs better than the state-of-the-art technique which achieved the best performance at the JNLPBA 2004.

As a follow-up study, we plan to improve the text mining technique where the context only option performs the worst. In several instances, we observe that it exacerbates the performance. Another research direction is to exploit various data sources, such as Gene Ontology (GO) and PharmGKB to study how an entity-specific dictionary could impact on the performance of entity extraction.

## Competing interests

The authors declare that they have no competing interests.

## Authors' contributions

MS conducted the statistical analyses and drafted the manuscript together with WSH and HJY. Both authors contributed to the design and conception of this work. All authors have read and approved the final version of the manuscript.
